# Recruitment interventions for trials involving adults lacking capacity to consent: methodological and ethical considerations for designing Studies Within a Trial (SWATs)

**DOI:** 10.1186/s13063-022-06705-y

**Published:** 2022-09-06

**Authors:** Victoria Shepherd, Fiona Wood, Katie Gillies, Abby O’Connell, Adam Martin, Kerenza Hood

**Affiliations:** 1 Centre for Trials Research, Cardiff University, 4th floor Neuadd Meirionnydd, Heath Park, Cardiff, CF14 4YS UK; 2 Division of Population Medicine, School of Medicine, Cardiff University, 8th floor Neuadd Meirionnydd, Heath Park, Cardiff, UK; 3 PRIME Centre Wales, School of Medicine, Cardiff University, 8th floor Neuadd Meirionnydd, Heath Park, Cardiff, UK; 4 Health Services Research Unit, University of Aberdeen, Health Sciences Building, Foresterhill, Aberdeen, UK; 5 Exeter Clinical Trials Unit, University of Exeter, Exeter, UK; 6 Academic Unit of Health Economics, Leeds Institute of Health Sciences, University of Leeds, Leeds, UK

**Keywords:** Recruitment, Informed consent, Mental capacity, Proxy, Research methodology, SWATs, RCTs, Dementia, Stroke

## Abstract

**Background:**

The number of interventions to improve recruitment and retention of participants in trials is rising, with a corresponding growth in randomised Studies Within Trials (SWATs) to evaluate their (cost-)effectiveness. Despite recognised challenges in conducting trials involving adults who lack capacity to consent, until now, no individual-level recruitment interventions have focused on this population. Following the development of a decision aid for family members making non-emergency trial participation decisions on behalf of people with impaired capacity, we have designed a SWAT to evaluate the decision aid in a number of host trials (CONSULT). Unlike in recruitment SWATs to date, the CONSULT intervention is aimed at a ‘proxy’ decision-maker (a family member) who is not a participant in the host trial and does not receive the trial intervention. This commentary explores the methodological and ethical considerations encountered when designing such SWATs, using the CONSULT SWAT as a case example. Potential solutions to address these issues are also presented.

**Discussion:**

We encountered practical issues around informed consent, data collection, and follow-up which involves linking the intervention receiver (the proxy) with recruitment and retention data from the host trial, as well as issues around randomisation level, resource use, and maintaining the integrity of the host trial. Unless addressed, methodological uncertainty about differential recruitment and heterogeneity between trial populations could potentially limit the scope for drawing robust inferences and harmonising data from different SWAT host trials. Proxy consent is itself ethically complex, and so when conducting a SWAT which aims to disrupt and enhance proxy consent decisions, there are additional ethical issues to be considered.

**Conclusions:**

Designing a SWAT to evaluate a recruitment intervention for non-emergency trials with adults lacking capacity to consent has raised a number of methodological and ethical considerations. Explicating these challenges, and some potential ways to address them, creates a starting point for discussions about conducting these potentially more challenging SWATs. Increasing the evidence base for the conduct of trials involving adults lacking capacity to consent is intended to improve both the ability to conduct these trials and their quality, and so help build research capacity for this under-served population.

## Background

Recruitment of participants into randomised controlled trials (RCTs) is essential for their successful conduct. However, trials involving adults with impaired capacity to consent are particularly challenging to conduct [1]. A recent review found that few trials are designed to include adults who lack capacity, even in populations that are particularly associated with cognitive impairment such as dementia and stroke, and the overall number of participants enrolled in trials who do lack decision-making capacity is worryingly low [2]. This contributes to a weak evidence-base available for the care of these populations. Improving access to research for groups who are under-served by research is one of the key areas for action in the UK government’s ‘Saving and Improving Lives’ plan for the future of clinical research delivery [3]. However, despite high profile initiatives to improve the inclusion of under-served populations in research, such as the NIHR INCLUDE programme [4], little attention has been paid to how the conduct of non-emergency trials involving adults lacking capacity can be improved. To date, individual-level recruitment and retention interventions have primarily focused on populations who are able to provide consent for themselves [5, 6] and children [7].

The growth in trials methodology research has largely been focused around interventions for increasing recruitment to trials [5, 8]. These are now being tested through embedding them in trials using ‘Study Within a Trial’ (SWATs) methodology, where participants in the host trial are randomised to different recruitment or retention processes within a trial [9]. SWATs are self-contained studies which are designed for synthesis across a number of host trials, and primarily involve the randomised or non-randomised evaluation of an intervention. They may additionally use qualitative methods to address questions about how a trial process is delivered [8]. Given the range of potential targets for improving trial conduct, the importance of prioritising interventions for evaluation in a SWAT has been highlighted [10]. One of the ‘top ten’ recruitment priorities from a recent SWAT prioritisation exercise (PRioRiTY I) was to establish the best approaches to ensure the inclusion and participation of under-represented or vulnerable groups in RCTs [11].

As part of a research programme addressing the methodological and ethical issues encountered in research involving adults with impaired capacity to consent, we have developed the first intervention to support proxy decisions about research participation [12]. Our decision aid for families making research decisions is now being evaluated as part of a SWAT programme (CONSULT) which will randomise family members to receive the decision aid alongside standard study information (intervention), or standard study information alone (control), in up to five host studies in a range of settings and populations [13]. An embedded process evaluation and cost consequence analysis, exploring the resources involved in intervention delivery, will enable the findings to be contextualised in order to draw robust conclusions about the effectiveness of the intervention and factors likely to affect implementation. The SWAT has been registered on the SWAT repository (SWAT #159 [14]).

Existing guidance for designing SWATs is not necessarily applicable to trials in populations where participants are unable to provide their own consent. For example, the guidance makes implicit assumptions that participants in the SWAT are also the individual who is receiving the intervention/comparator in the host trial [8]. The implications for a SWAT where this is not the case have not previously been explored. Similarly, ethical considerations to date have largely focused on whether individual participant consent is required for a particular SWAT and the process of seeking ethical approval [8]. More ethically complex questions about the implications of SWATs intended to enhance decisions about participation being made by another person, in addition to the ethical complexities surrounding proxy consent for research [15, 16], have yet to be addressed.

This commentary reports the main methodological and ethical considerations encountered when designing the CONSULT SWAT, with the aim of using this as a case study to inform future SWATs evaluating interventions for trials involving adults lacking capacity to consent in non-emergency settings.

## Methodological and ethical considerations for SWATs involving adults lacking capacity to consent

The methodological and ethical considerations are described in the following section. Table 1 provides a summary of the main considerations, together with our proposed solutions to address some of these issues.

**Table 1:** Summary of the main methodological and ethical considerations when designing SWAT in trials involving adults lacking capacity and proposed solutions

Methodological and/or ethical area	Description of the issue	Proposed solutions
Maintaining the integrity of the host trial	Impact on recruitment and retention rates from interventions aimed at improving proxy decision-making is unclear	• Undertake assessment of host trial context to ensure suitability for the SWAT and anticipate issues with embedding the intervention and/or SWAT • Adopt an exploratory approach to obtaining and analysing recruitment and retention data in host trials • Record and report factors affecting intervention effectiveness and/or implementation, and impact on the host trial
Identifying a suitable outcome measure	Far smaller amount of methodological research in trials involving adults lacking capacity, therefore less is known about appropriate outcomes and measurement instruments	• Factor in the need for preliminary work to establish relevant outcomes and outcome measurement instruments (including measurement timing) • Consider whether work is needed to develop or adapt (and validate) outcome measurement instruments prior to SWAT
Unpredictability of sample sizes	SWAT sample size is dependent on the host trials, which may be more heterogeneous and have greater uncertainty than for SWATs in other populations	• Work with the host trial team to assess the likely proportion of participants who will lack capacity (as a whole and by site) and the proportion expected to have a personal consultee/legal representative • Encourage reporting of capacity status and involvement of consultees/legal representatives in trials involving adults lacking capacity to inform future SWATs
Challenges in consent and data collection	SWAT participant is not generally a participant in the host trial and so does not usually provide their own consent or data for the host trial	• Incorporate flexibility into the design of the SWAT to enable alignment with host trial processes, and so minimise burden for trials and SWAT participants
Uncertainties about the resources needed to deliver the intervention	Trials involving adults lacking capacity are more resource-intensive and so determining the cost of delivering recruitment/retention intervention and conducting a SWAT is particularly important in these trials	• Explore how best to collect resource use data from proxies as non-participants in the host trial and from trial teams • Work is needed to disentangle the costs of delivering the intervention and SWAT from those needed to deliver the host trial • Additional work is needed to determine the most appropriate perspective for economic evaluations in these SWATs

### Maintaining the integrity of the host trial

One of the key requirements of a SWAT is that it must not reduce the scientific integrity of the host trial, its rationale, or measurement of outcomes [8]. Interventions such as the decision aid being evaluated in the CONSULT SWAT are intended to improve the experience of proxy decision-making for family members and to support them to make more informed decisions, rather than to influence recruitment and retention rates [12]. However, encouraging families (or other proxies) to carefully consider the question of participation through the use of the decision aid may influence recruitment to the host trial, in either a positive or negative direction. There may also be an effect on recruitment behaviour leading to differential recruitment [17], for example recruiters who have been trained in the decision aid may be more likely to approach people without capacity. Therefore, the methodological implications of having recruitment as an outcome of the SWAT are not clear.

There are similar uncertainties around the relevance of using retention as an outcome of the SWAT as although a more considered consent process might be expected to improve retention, the effect of interventions such as CONSULT on retention of participants in the host trial is unclear. For example, a participant enrolled in a host trial through proxy agreement may regain capacity and wish to withdraw from the trial. However, this may be due to various reasons including the practicalities of continued participation rather than reflecting the ‘quality’ or ‘informedness’ of the proxy’s consent decision. This means that whether the proxy’s decision is considered ‘good’ in terms of accurately reflecting the person’s wishes is extremely complex to untangle [16]. Whilst randomisation within the SWAT would likely balance these factors between the intervention and control arms, other factors affecting withdrawal (or loss to follow-up) for host trial participants might make it unlikely that an intervention effect can be discerned. However, due to the novel nature of the intervention, attempting to obtain and analyse retention data in host trials may be useful in terms of understanding the wider context and the relationships between proxy decisions and trial recruitment and retention.

Assessment of host trial context will be key to evaluating both the effectiveness of the intervention and implementation factors. Delivery of the decision aid may vary between host trials, as some trials will approach family members during a consultation, whereas others will recruit remotely by post or online with minimal or no contact between researchers and families. Therefore, establishing host trial recruitment processes and aligning the SWAT process will be an important part of assessing the feasibility of a trial being able to host the SWAT. Detailed records about the processes and any impact on recruitment in the host trial will be required in order to inform the evaluation of the intervention’s effectiveness and implementation.

### Identifying a suitable outcome measure

As there has been much less research on exploring proxy consent decisions, evaluating a novel intervention in this population has required considerable work to establish relevant outcomes and identify appropriate outcome measurement instruments. The primary outcome in the SWAT is decision quality as assessed through a recently developed scale (Combined Scale for Proxy Informed Consent Decisions – CONCORD) which measures domains such as the proxy’s understanding, values congruence, and satisfaction with their decision-making. Preliminary work has established the feasibility and validity of CONCORD, which will be concurrently validated in the CONSULT SWAT.

As with all similar interventions, perception of decision-making can be affected by biases such as recall or outcome bias; therefore, careful attention to the timing and impact of outcome measurement is needed. In the CONSULT SWAT, both the intervention and outcome measure are sensitive to the same constructs or concepts and so may be subject to question-behaviour effects whereby asking about decision-making behaviour produces small changes in the behaviour being asked about [18]. The CONCORD scale will be administered to proxies in both the control and intervention arms. This means that even SWAT participants allocated to the control arm will receive these ‘prompts’ which may affect their decision, regardless of whether they are aware of their allocation. For example, questions that ask whether their decision reflects the person’s wishes and preferences may influence their decision to agree to (or decline) participation if seen prior to making that decision or potentially prompt them to request the person’s withdrawal if they have already agreed to participation. Therefore, whilst outcome measure completion will be pragmatic and aligned with the host trial processes, it is expected that the CONCORD scale will be completed in a relatively short timeframe following the proxy decision.

### Unpredictability of sample sizes

The sample size of a SWAT is dependent on the host trial that it is embedded within [8]. Therefore, sample size calculations are not generally performed for SWATs [8] as the intention is to contribute to cumulated evidence [10]. For recruitment interventions in trials involving adults with cognitive impairment, only a proportion of potential participants will be assessed as lacking capacity to consent and so requiring the involvement of a consultee or legal representative as the proxy decision maker. This may vary considerably between trials and settings; for example, the proportion of patients lacking capacity to make decisions in some secondary care settings is around 34% [19], rising to around 70% in care homes [20] and over 90% in critical care [21]. However, few data are available on the proportion of potential participants who will lack capacity to consent to a trial, not least because capacity status is rarely reported in completed trials [2]. Therefore, predicting the number of host trial participants who would participate in this SWAT, and hence the feasibility of embedding the SWAT in a candidate host trial, may be more complex than in SWATs involving other populations. Conducting this SWAT and encouraging reporting of capacity status in relevant trials may help inform future SWATs in trials involving adults lacking capacity in non-emergency settings.

There will also be considerable heterogeneity in capacity status between populations, settings, sites, and between the proportion of potential participants who have a family member available and willing to act as personal consultee or legal representative. Our previous studies have found considerable variation between the use of personal consultees and legal representatives as opposed to professionals taking on the role, both between and within trials (e.g. across different sites) [2]. A SWAT aimed at family members as consultee/legal representative will therefore need to undertake detailed exploratory and feasibility work with the host trial team to assess the likely proportion of participants who will lack capacity as a whole and by site and the proportion who are expected to have a personal consultee/legal representative.

### Challenges in consent and data collection

A CONSULT SWAT participant is not generally a participant in the host trial unless, for example, the trial is also recruiting carers and so will not be receiving the intervention under investigation in the trial. Therefore, this raises additional considerations about the processes for obtaining consent and collecting baseline and follow-up data for individuals whose consent and data would not normally be obtained as part of the host trial.

In our recent qualitative study to explore the feasibility of conducting the SWAT, research teams expressed concern about the need to seek additional consent for the SWAT in an already complex consent encounter [22]. Therefore, in the CONSULT SWAT, rather than having separate consent documents, return of the completed questionnaire by SWAT participants will be taken as an indication of their consent to participate in the SWAT. Separate information and consent forms will be used for interviews in the process evaluation, and these will be administered by the core CONSULT SWAT management team to remove the burden from research teams at sites.

Family members who act as a consultee or legal representative and agree to the person they represent participating in a trial are usually asked to provide basic contact information. However, this would not usually include the demographic data that might be needed for a SWAT and information is not usually collected from family members who decline participation on the person’s behalf. Therefore, additional data collection pathways need to be introduced in the CONSULT SWAT and aligned with the host trial processes. If recruitment and retention data are being collected as part of the SWAT, an additional step will be required to link the SWAT participant to the host trial participant. This could potentially be through a recruitment log retained by the host trial, with only pseudonymised linked data provided to the SWAT team. As with all SWATs, data transfer agreements will need to be in place and clearly communicated to participants in the SWAT.

### Uncertainties about the resources needed to deliver the intervention

Economic evaluations are a core component of all phases of intervention research [23, 24], including RCTs to evaluate decision aids for treatment decisions [25]. However, there is currently a lack of guidance about how generally to conduct economic evaluations within SWATs. The economic evaluation in the CONSULT SWAT will be the first economic evaluation in a SWAT in trials with adults who lack capacity. It will take the form of a cost-consequence analysis and explore the implementation costs of the intervention and the resource use implications [26]. Resource use will include that incurred by the team (research costs) such as time to deliver the intervention and printing/postage costs where relevant and those incurred by the proxy as SWAT participant (participant costs) such as internet use if receiving the intervention remotely. Whilst some of these costs will be similar to those encountered in SWATs and trials involving participants who provide their own consent, trials involving adults lacking capacity are more resource-intensive [27] and involve more specialised members of research teams [22]. It is therefore important to determine the cost of conducting these studies because this could have implications for the affordability and decisions about whether or not to proceed with future SWATs in these populations. We anticipate needing to address methodological uncertainties, such as how best to collect resource use data from proxies (as non-participants in the host trial), disentangling the costs attributed to delivering the host trial from the costs of delivering the intervention and SWAT, and determining the most appropriate perspective for the economic evaluation.

### Selecting an appropriate randomisation strategy

Where a SWAT involves randomisation, it can sometimes be through the same process to that used for the host trial or may be a separate randomisation process [8]. However, unlike traditional SWATs, CONSULT participants are family members and not participants in the host trial and so randomisation will need to be entirely separate. Due to this, and the anticipated heterogeneity in trial designs, the level of randomisation to either the intervention or control arm will be dependent upon the host trial design. Randomisation will preferably be at an individual level (family member) as the intervention is considered to be highly amenable to randomisation at that level, but cluster randomisation (recruiter or site) may be required where the host trial itself is cluster randomised, or where cluster randomisation is the most feasible or least burdensome option for the host trial. The decision-making process and any reflections on the impact on the SWAT outcomes should be transparently reported.

In contrast to recruitment SWATs in other populations, there are more contact points between researchers and potential participants in trials involving adults with cognitive impairment. Research staff may need to visit on multiple occasions to provide information in a suitable format, assess capacity if required, and then identify and approach a family member to act as consultee or legal representative. Therefore, cluster randomisation at recruiter level may be complex, and there may be greater potential for contamination between intervention and control groups. The process evaluation embedded in CONSULT will attempt to capture any contamination and differential recruitment to the SWAT, as well as any adaptations which may undermine intervention fidelity.

### Considerations for analysis

In accordance with established SWAT methodology, our intention is to conduct a meta-analysis across host trials. As with all meta-analyses, judgements need to be made about whether it is sensible to combine studies [8]. Meta-analysis in the CONSULT SWAT will need to take account of the impact of different levels of randomisation and any differential recruitment. Due to a lack of prior evidence about proxy decision-making in a range of different contexts, the ability to meta-analyse across host trials is difficult to determine prospectively. The mixed methods process evaluation will enable the findings from the SWAT meta-analysis to be contextualised and the underlying theoretical approach to be refined in order to support future implementation.

## Conclusions

With the exception of a small number of paediatric studies, randomised SWATs to date have been conducted with populations who are able to provide their own consent to participate in the host trial and the SWAT when required. The development of a SWAT to evaluate a novel recruitment intervention for adults lacking capacity to consent has raised a number of methodological and ethical considerations that have not previously been encountered in SWATs. Explicating these challenges, and some potential ways to address them, creates a starting point for discussions about conducting these potentially more challenging SWATs as well as how to improve trials involving adults lacking capacity.

Some of the challenges we have outlined require further action by the research community to address. For example, the proportion of a trial population who are likely to lack capacity to consent is difficult to assess given the lack of available data. As part of the wider obligations to report the representativeness of trial populations, which is increasingly being required [28], future trials should consider collecting and reporting participant capacity status and the numbers recruited via alternative consent pathways. This SWAT only includes proxies making a prospective decision on behalf of someone who lacks capacity at the time of recruitment; however, additional ethical and practical challenges may be encountered in emergency research which is conducted without prior consent [29]. Gaining a better understanding about the recruitment of adults lacking capacity in emergency situations, alongside the potential learning from this SWAT, may lead to methodological interventions to support inclusion in emergency research. Similarly, the challenges encountered when members of the healthcare team are required to act as proxy decision-makers in situations where no family members are available also requires further exploration.

We hope that increasing the evidence base for the conduct of trials which involve adults who lack capacity to consent will lead to parity in trials methodology research focusing on these populations and help build research capacity in this area. It also takes us one step further towards ensuring this under-served population has equality in the opportunity to participate in research, and therefore to receive better evidence-based care in the future.

## Data Availability

Not applicable as no data were collected for this paper.
